# Multifocal pigmented villonodular synovitis coexisting in both the knee joint and the patella: a case report and literature review

**DOI:** 10.1186/s12891-017-1654-6

**Published:** 2017-07-06

**Authors:** Mingxuan Gao, Hong Li, Xiaoyan Liang, Xiaoyan Fu, Xusheng Li

**Affiliations:** 1 Department of Joint Surgery, Lanzhou General Hospital of PLA, NO. 333 Nanbinhe Road, Qilihe District, Lanzhou, Gansu Province 730050 China; 2 Ophthalmology Center, Lanzhou General Hospital of PLA, Gansu, 730050 China; 3 Ultrasonic Diagnosis Department, Lanzhou General Hospital of PLA, Gansu, 730050 China

**Keywords:** Pigmented villonodular synovitis, Giant cell tumor of tendon sheath, Localized form, Multifocal lesions, Patella, Popliteal fossa, Case report

## Abstract

**Backgroud:**

Pigmented villonodular synovitis (PVNS) is an uncommon entity of proliferative lesion of the synovium, presenting with different clinical signs and symptoms. PVNS rarely forms an osteolytic lesion in a bone. Here we report a unique case of PVNS with a nodular lesion in the left patella.

**Case presentation:**

A 37-year-old female was referred to our hospital with complaints of ongoing left knee pain and a painful and palpable mass in her left popliteal fossa. MRI demonstrated a nodular lesion in the left patella, diffuse affected synovial tissue in the left knee and an extra-articular mass in the left popliteal fossa. After a primary diagnosis of PVNS had been established, combined arthroscopic synovectomy and open resection were performed. The postoperative pathological diagnoses of the resected mass from the popliteal fossa, the affected synovial tissue and the lesion in the patella were consistent with PVNS. At 1-year follow-up, no evidence of recurrence was noted.

**Conclusions:**

Based on brief literature review of PNVS, we presented a very rare case of PVNS with a nodular lesion in the left patella, diffuse affected synovial tissue in the left knee and an extra-articular mass in the left popliteal fossa.

## Background

Pigmented villonodular synovitis (PVNS), which also has been known as tenosynovial giant cell tumor, is a rare proliferative lesion of the synovium [1, 2]. It has an incidence of 1.8 cases per million [3].The affected synovium of PVNS may process to invade and destroy the surrounding articular cartilage and bone [4–7]. However, no report has described a PVNS coexisting both in the knee and within the patella. In this case report, we present a unique PVNS case with a nodular lesion in the left patella, diffuse synovial lesion in the left knee and a localized mass in the left popliteal fossa.

## Case presentation

**Fig. 1:**
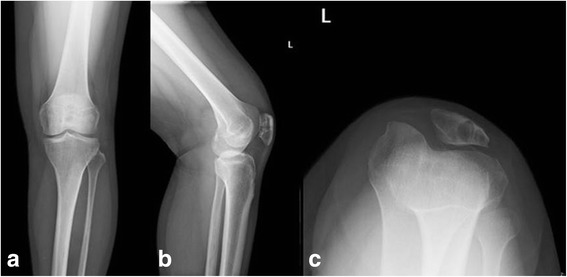
Frontal (**a**), lateral (**b**) and the axial (**c**) views of the patella show a well-defined lytic lesion in the left patella, which has eroded almost all the medial half of the patella. Small degenerative osteophyte formation and slight knee joint effusion is also presented

**Fig. 2:**
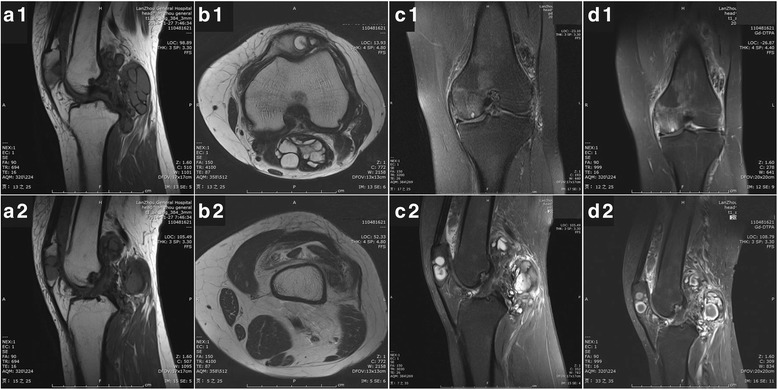
Magnetic resonance imaging (MRI) reveals extensive nodular synovial proliferation in the knee, a well-defined lesion confined to the patella and a lobulated mass-like lesion in the poplitea. Sagittal T1-weighted scans (*a1, a2*) show inhomogeneous low signal foci and depict a clear border of the foci. Axial T2-weighted scans (*b1, b2*) exhibits long T2 signal of joint effusion and tissue edema. Proton density-weighted imaging (*c1, c2*) shows lobulated, heterogeneous lesions with high intensity of PDWI signals are in patella, the suprapatellar bursa and the posterior region of knee. Focal hypointense areas represent the hemosiderin. No destructive lighment changes and cartilage damage is detected. After the administration of gadolinium-DTPA fat suppressed T1-weighted imaging (*d1, d2)* illustrates a distinct enhancement of the foci. The MRI appearance of is suggestive of the PVNS diagnosis

A previously healthy 37-year-old female was referred to our hospital with complaints of a 3-year history of intermittent left knee pain. The pain could be aggravated by physical activity such as long standing or walking. An egg-size mass in her left popliteal region was unintentionally palpated half a year ago. There was no history of trauma. She did not experience any other obvious symptoms or any locking episodes. Physical examination of the left knee revealed slight joint effusion and tenderness along joint line. A painful soft tissue mass was distinctly palpated in her left popliteal fossa. The range of motion was restricted in between 10 and 115 degrees because the pain would be exacerbated by deep extension or flexion. No positive sign of knee instability was induced by performing the Lachman test, the anterior and posterior drawer tests [8]. Plain radiographs (Fig. 1) showed a well-defined osteolytic lesion in the left patella. The lesion was larger than 2 cm. The medial half of the patella was almost eroded by the lesion. Small degenerative osteophyte formation and slight knee joint effusion was also presented (Fig. 1). Magnetic resonance imaging (MRI) of the left knee revealed extensive nodular synovial proliferation in the knee, a well-defined lesion confined to the patella and a lobulated mass-like lesion in the poplitea. Heterogeneous low signal foci with clear border was depicted on T1-weighted scans. Axial T2-weighted scans exhibited long T2 signal of joint effusion and tissue edema. Proton density-weighted imaging showed lobulated, heterogeneous lesions with high intensity of PDWI signals are in patella, the suprapatellar bursa and the posterior region of knee. Focal hypointense areas represented the hemosiderin. No destructive ligmental changes and cartilage damage were detected. After the administration of gadolinium-DTPA fat suppressed T1-weighted imaging illustrated a distinct enhancement of the foci. The MRI appearance of was suggestive of the PVNS diagnosis (Fig. 2).

**Fig. 3:**
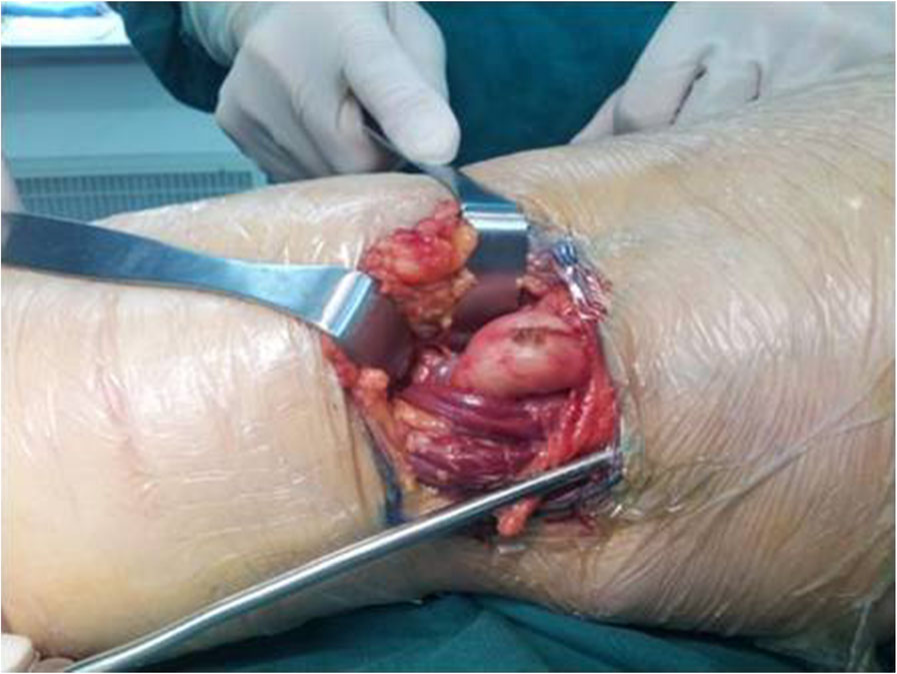
A short S-shaped incision was mad over the posterior knee joint. The posterior mass in poplitea was exposed, dissected and marginally resected

Combined arthroscopic total synovectomy, open resection of lesions, and iliac crest bone graft for patellar bone defect were performed under a general anaesthetic. The patient was in the prone position and a short S-shaped incision of dorsal approach was made for excision of the extra-articular mass in popliteal fossa [9].The mass showed brown in colour and had a thin pedicle firmly adhering to posterior knee capsule (Fig. 3). The mass conceivably communicated with the affected synovium of the intra-articular knee joint via the pedicle. The resected mass was a bit hardly elastic and oval lesion measuring 3.0 × 2.5 × 5.5 cm. A piece of tissue from the resected mass was taken for frozen section and the clinical diagnosis of PVNS was confirmed by the rapid pathological examination.

**Fig. 4:**
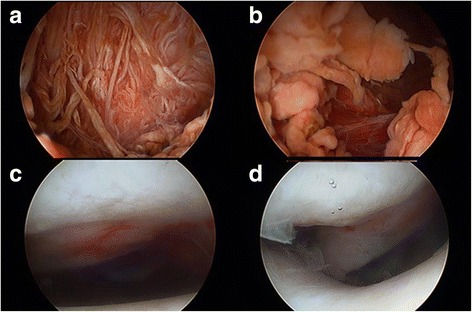
Arthroscopic images (**a**, **b**) of the left knee indicating prolific synovium with coarse villi, heavily pigmented diffuse pigmented villonodular synovitis. No communicating link was found under arthroscopic observation (**c**). Although the chondromalacia changes are visible on the articular surface of patella, it seems exactly intact without any eroded hole (**d**)

Then, the patient was turned over in supine position. Arthroscopic synovectomy was done using standard anterolateral, anteromedial and posteromedial portals to ensure maximum removal of affected synovium [10, 11]. Dark brown bloody fluid drained from the joint with introduction of arthroscopy cannula. Synovium was hypertrophic with brownish villi formation. Widely spread affection of synovium noted. Posterior compartment of the knee was having maximum amount of hypertrophic Synovium. The synovial lesion was resected and treated with shaver to achieve a macroscopic complete removal of PVNS. The synovial bed was cauterized with the radio frequency abrasion to stop bleeding and to reduce the chances of recurrence. Careful examination of the whole knee was done again. Macroscopically, no residual lesion or affected synovium tissue was left. There were only slight chondromalacia changes (Fig.4 c, d) visible on the articular surface of patella, but no eroded hole going through from intra-articular space into the lesion of the patella was found. We did not clarify any direct connective tissue between the lesion in the patella and that of the knee. The menisci and cruciate ligaments were all good.

**Fig. 5:**
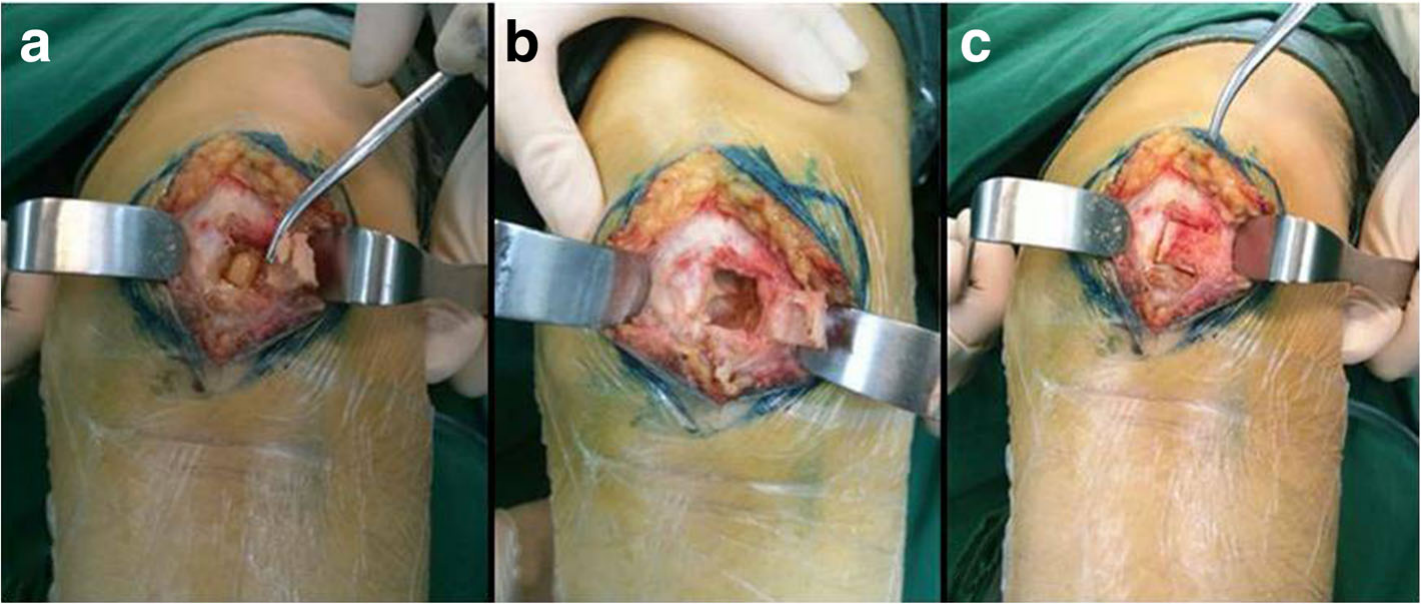
A cortical window of 1.5 × 1.5 cm was made over the anteromedial aspect of the patella (**a**). The tumor was brownish (**a**) and was scooped out. On the inner side of the cavity there is a sclerotic margin (**b**). Bone defect in the patella was filled with iliac crest bone graft (**c**)

Addressing to the lesion in the left patella, extended curettage was contemplated with the procedures as described by Kundu et al. [12]. A cortical window of 1.5 × 1.5 cm was made over the anterior patella. The bulk of the tumor was scooped out (Fig.5 a, b). The tumor was macroscopically in the same colour as that resected from the popliteal region and it was kept for routine histopathological examination. On the inner side of the cavity there was a sclerotic margin, which was treated with a high-speed burr until cortical bony surface with punctate visible bleeding. Next, the 50% zinc chloride was used as an adjuvant to chemically “burn” the tumor cavity [13, 14]. After that, the cavity was further washed with hydrogen peroxide. Nooks and corners were treated carefully to leave no macroscopic disease anywhere in the cavity. Bone defect in the patella was filled with iliac crest bone graft (Fig.5 c). Closure in layers and negative suction drain was done.

**Fig. 6:**
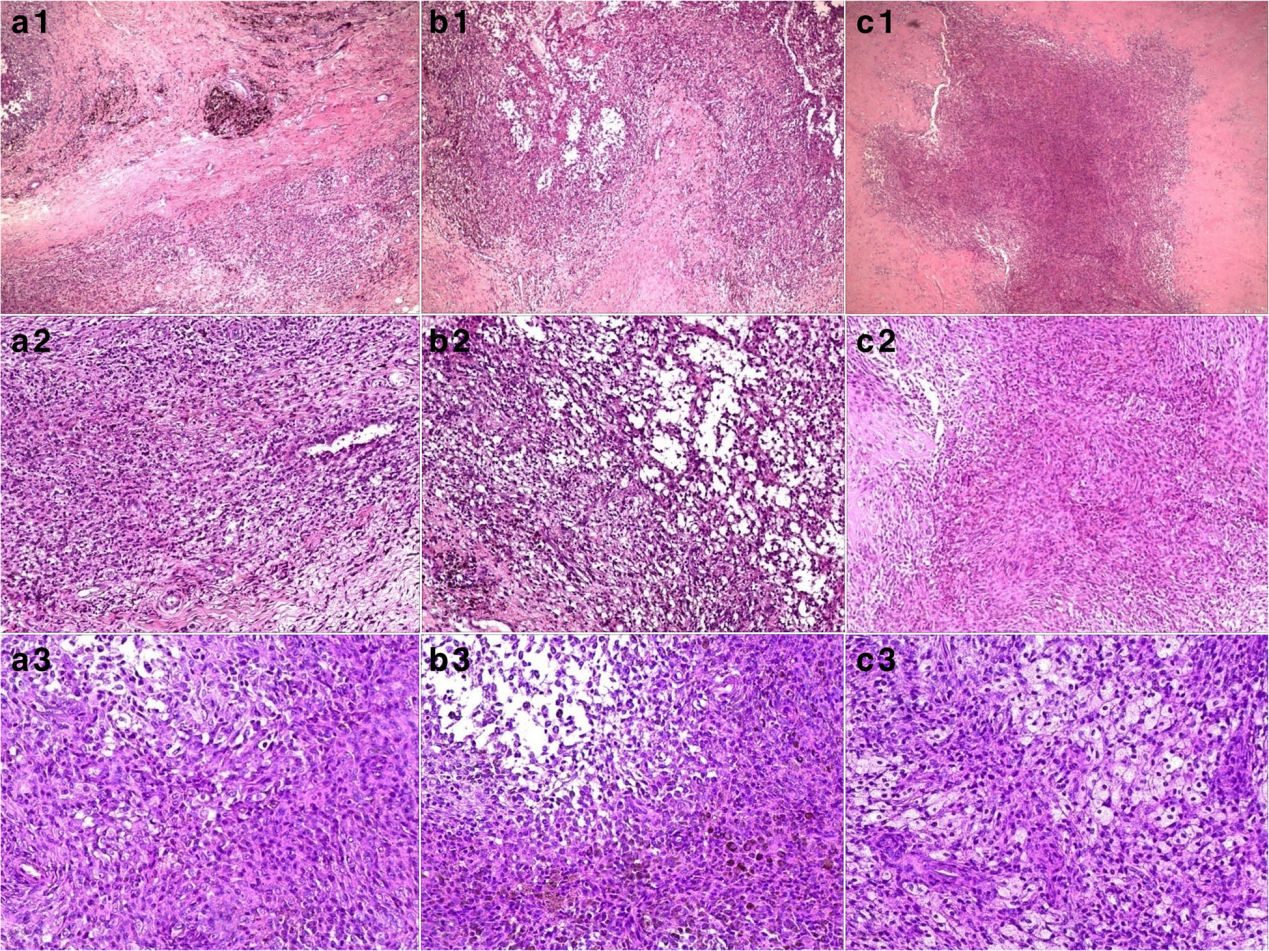
Photomicrographs of H&E stain samples, which were taken from the resected mass in poplitea (a1–3), the proliferated synovial tissue in the knee (b1–3) and the lesion in the patella (c1–3), respectively. Low power views (a1, b1, c1; ×40) are characterized by a prominent number of epithelioid cells with fibrous stroma. Medium power (a2, b2, c2; ×100) views demonstrates numerous mononuclear cells, scattered multinucleated giant cells and occasional cytoplasmic hemosiderin. Under higher magnification (a3, b3, c3; ×200), the mononuclear cells are round or spindled and they have minimal cytoplasm and eccentric nuclei. There are some foam cells. Some cells typically contain cytoplasmic hemosiderin granules. No mitotic figures and atypia were found. The pathological features of the lesions from different locations are very similar to one another. Histologically, all findings consistently confirmed the original diagnosis of PVNS

She tolerated the surgical treatment very well. The postoperative clinical course was uneventful. All tissue samples taken from the resected mass in popliteal fossa, arthroscopically from the intra-articular knee or from the lesion in the patella were definitively consistent with the PVNS diagnosis (Fig. 6). Cefazolin sodium 30 mg kg^−1^ per dose every 6 h were utilized from half an hour pre-operatively to 24 h post-operatively to prevent infection. Range motion exercises were started as pain decreased. Suture removal was done on day 12. Adjuvant external beam radiation [15, 16] was used from day 15. A total of 20 Gy delivered in 10 sessions every 2 days, with a duration of 20 days. One year postoperatively the patient was asymptomatic, with a full range of motion. No evidence of recurrence was noted.

## Discussion

Pigmented villonodular synovitis (PVNS) is a rare disorder, involving proliferation of the synovium of a joint, mucosal bursa, tendon sheath [1–3]. Typically, patients are adults in the third or fourth decade of their life [1]. Patients often present with complaints of joint pain and swelling [1, 17].The excessive bleeding of the affected synovial tissue into the joint space could cause suddenly extreme pain. The lesions of PVNS are almost exclusively benign with rare reports of malignancy [18–20]. The aetiology of this disease entity still remains unclear [1, 7, 18]. In 1941, Jaffe et al. recognized the common histological appearance of this disease characterized by lipid laden macrophages, multinucleated giant cells, and deposits of hemosiderin within a fibrous stroma. They created the term PVNS to describe this rare disease with proliferative, villonodular lesion of synovial membranes [21]. However, whether PVNS is an inflammatory lesion or a neoplasm has been controversial for a long time, and the cells of origin have not been elucidated. Growing observations of trisomy, and clonal DNA rearrangements as well as some rare descriptions of malignant transformation supported that its aetiology was neoplastic [18–20]. Over expression of colony-stimulating factor 1 (CSF-1) also suggests that PVNS is a true neoplasm [22, 23]. In contrast to the supporters of a neoplastic origin of the disease, the theory of inflammatory process is also a partly accepted pathogenesis by some researcher. Sakkers et al. [24] demonstrated polyclonality of the cell population in a patient with histologically proven PVNS and Oehler et al. [25] found positive staining for cell markers of inflammation.

Based on the growth pattern and clinical behaviour, in 1976, Granowitz et al. [26] roughly differentiated PVNS into two types, localized and diffuse. The diffuse type usually involves the entire synovial membrane of large joints, like the knee and the hip, whereas localized type mostly occurs around the fingers. Localized type is generally indolent, but the diffuse type is more aggressive. Localized type is with a minimal rate of recurrence after removal, but diffuse type is a highly recurrent form with the capacity of eroding adjacent bone and soft tissue [11, 21].The knee was reported the most affected joint accounting for up to approximately 75% of the cases of diffuse type [1, 21].Which type should this present case be categorized into, diffuse or localized? If the disease were confined to the intra-articular space of knee, it would be easily regarded as diffuse type. However, the current case has another two isolated lesions. Especially, the one in the patella is more like localized type. We did not find any similar report in the previous literature.

There have been some publications of cases with bifocal or even multifocal PVNS involvement [2, 27]. The disease may involve the same joint in both extremities or even several different joints in a patient [28, 29].The involved locations of localized PVNS arising from knee have been reported. It can be the anterior cruciate ligament, posterior cruciate ligament, or patella fat pad [2, 30–32]. The lesion in the popliteal fossa, like the present case, is usually thought as the extra-articular extension of the intra-articular PVNS. The affected synovium of PVNS may lead to erosive destruction of the surrounding articular cartilage and bone [33]. However, bone erosions are only occasionally seen in the joints usually affected by the disease. The involved bone of the reported cases includes femoral head, femoral condyle, tibial epiphysis, humeral head, and talus [4]. However, in no previous case the disease has been published to be with both intra-articular diffuse lesion of knee and nodular lesion in the patella as well. During the operation of our case, we did not identify any direct communication between the lesion in the patella and those of the knee. It was hard to be sure whether the tumor in the left patella originated from that of the left intra-articular knee or not. It also could not be explained as the result of the affected synovium invading the patellar surface and forming a lesion in the patella, because the articular surface seems exactly intact under arthroscopic observation (Fig. 4). However, the post-operative histology shows all the same diagnosis of PNVS (Fig. 6). We speculated that the lesion in the left patella came from the adjacent affected synovium of the knee joint somehow. Because symptoms of PVNS develop gradually, our patient sought medical support after progressive complaints over months to years. The interval between the onset of symptoms and definitive diagnosis was more than 3 years. Therefore, the delay in management possibly was the only clue implying that the lesion in the patella had a relation with the lesion in the knee.

The diffuse type of PVNS recurrence is always a challenge, which was reported to be as high as 50% when complete resection of affected synovium is not achieved [16, 34]. Arthroscopic resection has grown to be a valuable option for PVNS arising in the knee joint [35–37]. Arthroscopy offers the advantages of direct visualization and easy accessibility. One major postoperative complication of open approaches is the occurrence of joint stiffness. Less invasive procedures using arthroscopic approaches decrease the rate of this morbidity. With standard arthroscopic technique of radical synovectomy, the recurrence could be reduced to rates as low as 8 to 18%. In our case, the synovial lesion was resected under arthroscopy. However, the extra-articular foci in the patella and the popliteal fossa had to be addressed by open approaches. Previously published studies have shown the 50% zinc chlorid is effective on giant cell tumor of bone (GCTB) [13, 14]. In this case, PVNS of patella was locally aggressive and osteolytic. The biological behaviour is very similar to GCTB. We tried zinc chlorid as an adjuvant to chemically treat tumor cavity. However, PVNS and GCTB are two completely different entities and they can not be confused with each other. Further studies are still necessary to approve the effect of 50% zinc chlorid on PVNS of bone.

Many studies have shown that peri-operative radiotherapy for PVNS is associated with a low rate of recurrence, and that external beam radiation is a safe and effective adjuvant therapy for PVNS [15, 16]. The risk of radiation-induced cancer due to radiotherapy for PVNS was estimated very small [38]. Under arthroscopy, the intra-articular lesion in the knee looked more like a diffuse PVNS, which protruded into the popliteal region and formed a mass there. These signs showed the lesion in knee was, to some extent, active and invasive. It was very difficult to draw a clear line between affected synovium and intact area with naked eyes under arthroscopy. It was highly possible that the lesion in knee could not be absolutely excised. Taken together, we post-operatively prescribed external beam irradiation therapy for this patient to reduce the possibility of recurrence.

The 1-year follow-up showed no sign of relapse, but it is too early to say it would not reoccur sometime because PVNS has a high recurrence rate in the first post-operative 5 years [1, 16, 34]. When the recurrence is suspected, MRI can be used to detect. Once it is certain, revision surgery will be necessary.

## **Conclusions**

In conclusion, this is the first report of PVNS with a nodular lesion in the left patella, diffuse affected synovial tissue in the left knee and an extra-articular mass in the left popliteal fossa. The aim of this case report is to increase the awareness of bone and extra-articular involvement of PVNS.

## **Abbreviations**

CSF-1: Colony-stimulating factor 1
MRI: Magnetic resonance imaging
PVNS: Pigmented villonodular synovitis
